# Sex hormone-binding globulin (SHBG) mitigates ER stress and improves viability and insulin sensitivity in adipose-derived mesenchymal stem cells (ASC) of equine metabolic syndrome (EMS)-affected horses

**DOI:** 10.1186/s12964-023-01254-6

**Published:** 2023-09-11

**Authors:** Nabila Bourebaba, Mateusz Sikora, Badr Qasem, Lynda Bourebaba, Krzysztof Marycz

**Affiliations:** 1 Department of Experimental Biology, Faculty of Biology and Animal Science, Wrocław University of Environmental and Life Sciences, Norwida 27B, 50-375 Wrocław, Poland; 2 Department of Medicine and Epidemiology, UC Davis School of Veterinary Medicine, Davis, CA 95516 USA; 3 Department of Veterinary Medicine and Epidemiology, Veterinary Institute for Regenerative Cures, School of Veterinary Medicine, University of California, Davis, CA USA

**Keywords:** EMS, ASC, SHBG, ER Stress, Apoptosis, PDIA3, Insulin resistance

## Abstract

**Background:**

Equine metabolic syndrome (EMS), which encompasses insulin resistance, low-grade inflammation and predisposition to laminitis is a critical endocrine disorder among the most prevalent conditions affecting horses from different breeds. According to the most recent research, low human sex hormone-binding globulin (SHBG) serum levels correlate with an increased risk of obesity, insulin resistance and diabetes, and may contribute to overall metabolic dysregulations. This study aimed to test whether exogenous SHBG could protect EMS affected adipose-derived stromal stem cells (EqASC_EMS_) from apoptosis, oxidative stress, ER stress and thus improve insulin sensitivity.

**Methods:**

EqASC_EMS_ wells were treated with two different concentrations (50 and 100 nM) of exogenous SHBG, whose biocompatibility was tested after 24, 48 and 72 h of incubation. Several parameters including cell viability, apoptosis, cell cycle, reactive oxygen species levels, ER stress, Pi3K/MAPK activation and insulin transducers expression were analysed.

**Results:**

Obtained data demonstrated that exogenous SHBG treatment significantly promoted ASCs cells proliferation, cell cycle and survival with reduced expression of p53 and p21 pro-apoptotic mediators. Furthermore, SHBG alleviated the oxidative stress caused by EMS and reduced the overaccumulation of intracellular ROS, by reducing ROS + cell percentage and regulating gene expression of endogenous antioxidant enzymes (Sod 1, Cat, GPx), SHBG treatment exhibited antioxidant activity by modulating total nitric oxide (NO) levels in EMS cells as well. SHBG treatment dampened the activation of ER stress sensors and effectors in EqASC_EMS_ cells via the upregulation of MiR-7a-5p, the decrease in the expression levels of ATF-6, CHOP and eiF2A and the restoration of PDIA3 chaperone protein levels. As a consequence, SHBG application substantially improved insulin sensitivity through the modulation of Pi3K/Akt/Glut4 insulin signalling cascades.

**Conclusion:**

Our results suggest that the SHBG is endowed with crucial beneficial effects on ASCs metabolic activities and could serve as a valuable therapeutic target for the development of efficient EMS treatment protocols.

Video Abstract

**Supplementary Information:**

The online version contains supplementary material available at 10.1186/s12964-023-01254-6.

## Background

Equine metabolic syndrome (EMS) is a more and more prevalent and devastating endocrine disorder affecting horses all over the world [1]. Horses that suffer from EMS are characterized by a constellation of physiological compilation that when not treated often lead to laminitis development – a life-threatening disorder. EMS is characterized by specific clinical features, including regional adiposity in the neck, tail head, and periorbital area, a history of or persistent laminitis, and insulin resistance. EMS is caused by a combination of two critically important factors that include lack of physical activity combined with high carbohydrates intake; in the majority of horses, it is leading to obesity development although obesity per se is not required for the clinical diagnosis of EMS [1]. It is estimated that between 19 and 40% of the horse population are affected by obesity; notably, in the United Kingdom, the rate of obesity surpasses 40% in the equine population. Together with obesity horses affected by EMS are characterized by abnormal insulin response to oral glucose (often hyperinsulinemia), hyperleptinemia, local and systemic inflammation, hypertriglyceridemia, or mild triglyceridemia and dyslipidemia [2]. In the course of EMS liver, an adipose tissue axis is strongly believed to play a critical role in disease progression. It was previously shown, that at the molecular level hepatocytes suffer from progressive apoptosis, increased endoplasmic reticulum, oxidative stress, excessive accumulation of lipids, increased fetuin, and inflammation [3]. Therewith, oxidative stress results from an imbalance between reactive oxygen species (ROS) and antioxidants. In EMS, obesity and abnormal fat release pro-inflammatory molecules, weakening antioxidant defenses and causing oxidative stress. Insulin resistance can also lead to mitochondrial dysfunction and chronic inflammation, further contributing to ROS production. Indeed, a study conducted by our group in which Marycz et al*.* investigated how EMS impacts viability, senescence, and stress factors of EqASCs revealed that at the cellular level, p53 directly activates BAX to permeabilize mitochondria, leading to DNA damage, ROS accumulation, and cytochrome c release. This induces caspase activation and finally apoptosis [4]. On the other hand, several studies have demonstrated that endoplasmic reticulum stress (ER Stress) is a central feature implicated in the pathogenesis of insulin resistance and type 2 diabetes, which are linked to metabolic syndrome. According to a recent study conducted by Foss-Freitas et al*.*, carried out on patients with Familial partial lipodystrophy of the Dunnigan type (FPLD) to shed light on the link between metabolic abnormalities, the inflammatory profile and the expression of genes involved in the activation of ER stress with FPLD; the results demonstrated that an accumulation of stress stimuli including ROS, lipid peroxides and damaged DNA induce serious alterations of cellular proteins and a general proteostasis disruption, that participates to the initiation of an unfolded protein response (UPR). As a result, the ER-associated UPR sensors undergo a series of molecular cascades to support a cellular adaptative response that culminate with a reduction in protein synthesis pathways and a programmed death occurrence, when cells lose their ability to properly regulate the proteostatis status [5].

The unfavorable microenvironment of adipose tissue of EMS horses affects the same time adipose-derived mesenchymal stem cells (ASC) residing within which limits their therapeutic potential. ASC exhibit the presence of specific surface markers that includes CD90^+^, CD105^+^, CD44^+^ and lack expression of CD45^−^, as well as enhanced activity of multipotency transcripts by the expression of transcription factors including Octamer Binding Transcription Factor‐4 (Oct4), Sex Determining Region Y Box‐2 (SOX2) and Nanog Homeobox Protein (Nanog), that modulates their proliferative and self-renewal potential. The high proliferative potential, viability, and ability to self‐renew and differentiation potential are the most important features of ASCs from a clinical perspective [6, 7]. ASC are multipotent stem cells with the ability to secrete a wide plethora of so-called pro-regenerative factors which include vascular endothelial growth factor (VEGF), bone morphogenetic protein (BMP‐2), or fibroblast growth factor (FGF) makes them critically important as a cytological modulator of inflamed and insulin-resistant adipocytes. The author’s previous study showed that systemic administration of rejuvenated ASC in EMS horses stimulates regulatory lymphocyte activity (Tregs) which leads to decreased systemic inflammation through the improvement of liver function [8]. However, our previous results showed, that in vitro pre-treatment of ASC before their clinical application is strongly required to observe the positive clinical effect. This conclusion is based on several years of extensive research done by our group in which we showed that ASC isolated from EMS horses seriously suffer from decreased proliferative potential, increased apoptosis and senescence, abundant oxidative stress, and finally mitochondrial metabolism and dynamics impairment [9, 10]. Moreover, abnormal DNA methylation combined with mitochondrial autophagy impairment might be a key component that induces ER stress leading to insulin resistance development.

Sex hormone–binding globulin (SHBG), also known as the sex steroid-binding protein (SBP), is a 90–100 KDa homodimeric glycoprotein that is encoded by the gene of chromosome 17, and as recently shown is produced by hepatocytes and adipocytes [11, 12]. SHBG as an extracellular plasma glycoprotein that binds to circulating steroid hormones, including testosterone, dihydrotestosterone, and oestradiol, reducing their metabolic clearance rate and modulating their bioavailability and access to target tissues and cells [13]. Moreover, it was shown that ligand-bound SHBG binds to membrane receptors, and stimulates cAMP production, and/or enters cells by binding to the membrane protein megalin to initiate a biological effect [14]. SHBG systemic level has been shown to be decreased in patients with obesity, type 2 diabetes (T2DM), and in patients with metabolic syndrome (MetS) [15–17]. Our unpublish data confirms that findings since we have found that EMS mares are affected by lowered SHBG levels, depending on part of the year. Recent, promising findings indicate that SHBG protects against oxidative stress, and ER stress development, through decreased expression of inositol-requiring enzyme 1 (IRE1α), DNA damage-inducible transcript 3 (CHOP), activating transcription factor 6 (ATF6), and immunoglobulin heavy chain-binding protein (BIP). Little is known, however, how SHBG affects ASC oxidative stress, ER stress and finally whether SHBG might modulate insulin sensitivity.

In this study, we were interested in whether SHBG modulates ASC isolated from EMS horse’s apoptosis, ER stress, oxidative stress and insulin sensitivity. We have found that SHBG by mitigation of apoptosis, ER stress, oxidative stress and insulin-sensitizing activity might be considered in the future as therapeutic agent for EMS treatment.

## Materials and methods

### Isolation and propagation of EqASC

Equine ASC cells (HE and EMS) were obtained from the cell collection of the Department of Experimental Biology, University of Environmental and Life Sciences, Wrocław, Poland. Cells were suspended in a complete growth medium (CGM) that consisted of Dulbecco′s Modified Eagle′s Medium—low glucose (containing 1000 mg/L of glucose) supplemented with 5% of foetal bovine serum (FBS) and 1% of P/S. The cells were maintained at standard conditions (37 °C, 5% CO2 and 95% humidity). After reaching ~ 90% of confluency the cells were passaged using Trypsin–EDTA solution [18].

### Treatment of EqASC cells with SHBG protein

The isolated EqASCs were seeded on 24-well dishes at the density 30 000 cells / well (the analysis performed during this study had been replicated four times). Simultaneously, the SHBG protein was prepared accordingly to manufacturers indications (SHBG native protein, Fitzgerald, 30R-AS012, 1 mg). The protein was reconstituted in sterile PBS (phosphate buffered saline) to obtain the concentration of 1 mg/mL. Moreover, 4% solution of sterile BSA in distilled water was prepared. After reaching approximately 80% of confluency the cells were washed with HBSS and fresh DMEM Low Glucose medium with 5% addition of previously prepared BSA was added to the cultures. The media did not contain FBS or P/S in order to eliminate potential confounding factors that may interfere with the results of the experiment. SHBG in two concentrations of 50 nM and 100 nM was added to experimental groups. The experiment was finished after 24 h of SHBG protein treatment. The experimental plan included 4 groups: EqASC_HE_, EqASC_EMS_, EqASC_EMS_ 50 nM SHBG and EqASC_EMS_ 100 nM SHBG.

### Analysis of EqASC metabolic activity

The metabolic activity of EqASC cells were measured using well-established MTS assays. For this purpose, the cells were washed with HBSS and 500 µL of CGM with 10% addition of MTS dye (ab197010, Abcam) was added. Next, the cells were incubated for 2 h in CO_2_ incubator in standard culture conditions. Then, the supernatants were transported to 96-well dish. Afterwards, the absorbance was measured at 490 nm wavelength.

### Evaluation of cellular cytotoxicity

Pierce™ LDH Cytotoxicity Assay Kit (88,953, Thermo Fisher Scientific) was used in order to evaluate the cytotoxicity of LDH protein after incubation with SHBG. The assay was performed accordingly to manufacturer protocol. After incubation with SHBG protein 50 µL of medium from each experimental group was transferred to new 96-well dish in triplicate. Additional wells were dedicated for: LDH background (serum-free medium), spontaneous LDH activity control (water) and maximum LDH activity control (10 × lysis buffer). After 45 min of incubation in standard culture conditions, 50 µL of Reaction Mixture was added to each well and incubated for 30 min at room temperature in the dark. Subsequently, 50 µL of Stop Solution was added to each well and gently mixed. The plate was measured spectrophotometrically at 490 nm and 680 nm wavelength. The absorbance was measured using the formula $A\left(480-680\right)=LDH 480nm-LDH 680nm$. The % of cytotoxicity was measured using the formula:

$$\% Cytotoxicity=\frac{\left(SHBG-treated LDH activity\right)-(Spontaneous LDH activity) }{\left(Maximum LDH activity\right)-(Spontaneous LDH activity) } \times 100\%$$

### Cytometric evaluation of viability and apoptosis profile

The analysis of apoptosis profile of EqASC_HE_, EqASC_EMS_, EqASC_EMS_ 50 nM SHBG and EqASC_EMS_ 100 nM SHBG was carried out using Muse™ Annexin V & Dead Cell Kit (Luminex; cat. no.: MCH100105, Poznań, Poland). The procedure was performed accordingly to manufacturer’s instruction and described previously [19]. The cells were trypsinized (Trypsin–EDTA solution, T3924, Sigma Aldrich, Poznan, Poland) and resuspended in 100 µL of PBS (phosphate-buffered saline) with the 1% addition of FBS. Then, 100 µL of Muse™ Annexin V & Dead Cell Reagent was added to the cells and the samples were incubated for 20 min in the dark at room temperature. The dye provided by the producer consists of two dyes: Annexin V and 7-Aminoactinomycin D (7-AAD) that separate the cells population into four sub-populations: viable cells, early apoptotic cells, late apoptotic cells and dead cells. After incubation, the cells were analysed using Muse™ Cell Analyzer.

### Cytometric evaluation of cells’ cycle

The analysis of cell cycle was investigated using Muse™ Cell Cycle Kit (Luminex; cat. no.: MCH100106, Poznań, Poland). The procedure was performed accordingly to manufacturer’s instruction and described previously [20]. The cells were trypsinized (Trypsin–EDTA solution, T3924, Sigma Aldrich, Poznan, Poland) and washed once with PBS. Then, 200 µL of ice-cold 70% ethanol was slowly added to the samples and incubated for 12 h at -20 ˚C. Afterwards, the cells were centrifuged (5 min, 300 × g) and washed once with PBS. Then, 200 µL of Muse™ Cell Cycle Reagent was added to the cells and incubated for 30 min in the dark at room temperature. The dye provided by the manufacturer consists of two reagents: RNAse A and propidium iodide (PI) that separate the cells population into three sub-populations: G0/G1, S and G2/M. After incubation the cells were analysed using Muse™ Cell Analyzer.

### Cytometric evaluation of oxidative stress

The analysis of oxidative stress was measured using accumulation of reactive oxygen species (ROS) and nitric oxide (NO). The ROS accumulation was analysed using Muse™ Oxidative Stress Kit (Luminex; cat. no.: MCH100111, Poznań, Poland). The procedure was performed accordingly to manufacturer’s instruction and described previously [20, 21]. The cells were trypsinized (Trypsin–EDTA solution, T3924, Sigma Aldrich, Poznan, Poland) and 10 µL of cells were added to 190 µL of Muse™ Oxidative Stress Working Solution and incubated 30 min at 37 ˚C. The Working Solution is based on dihydroethidium (DHE) that separate the cells population into two sub-populations: ROS- and ROS + cells. After incubation the cells were analysed using Muse™ Cell Analyzer.

For the purpose of nitric oxide analysis, the Muse™ Nitric Oxide Kit (Luminex; cat. no.: MCH100112, Poznań, Poland) was used. The procedure was performed accordingly to manufacturer’s instruction. After trypsinization 10 µL of cells were mixed with 100 µL of Muse Nitric Oxide Working Solution and incubated 30 min at 37 ˚C. Then, 90 µL of 7-AAD Working Solution was added to the samples. The dyes are based on DAX-J2 Orange dye and 7-AAD dye that separate the cells population into four sub-populations: viable cells with NO, viable cells without NO, dead cells with NO, dead cells without NO. The cells were analysed using Muse™ Cell Analyzer.

### Cytometric evaluation of PI3K/MAPK pathway dual activation

The activation of PI3K/MAPK pathway was measured using the Muse™ PI3K/MAPK Dual Pathway Activation Kit (Luminex; cat. no.: MCH200108, Poznań, Poland). Briefly, the cells of each group were trypsinized (Trypsin–EDTA solution, T3924, Sigma Aldrich, Poznan, Poland) and fixed using Fixing Buffer for 10 min on ice. After washing, the cells were permeabilized using a Permeabilization Buffer for 10 min on ice. A number of 200,000 cells were subsequently transferred to 90 µL of Assay Buffer and mixed with 10 µL of phospho-specific Akt (Ser473)-Alexa Fluor™ 555 and a phospho-specific ERK1/2 (Thr202/Tyr204, Thr185/Tyr187)-PECy5 conjugated antibodies Cocktail. The cells were incubated for 30 min in the dark at room temperature. After washing using the provided Assay Buffer, cells were resuspended in 200 µL of Assay Buffer and analysed using a Muse™ Cell Analyzer.

### Confocal microscopy

After 24 h of cells treatment with SHBG protein the cultures were stained for confocal microscope analyses. The ROS accumulation was stained using Reactive Oxygen Species (ROS) Detection Reagents (Invitrogen, CM-H2DCFDA) characterized by excitation / emission values of 492–495 nm / 517–527 nm respectively. The reagent was reconstituted in DMSO following the manufacturer protocol and added to the cells at a final concentration of 10uM. The cells were incubated 30 min at 37 ˚C in CO_2_ incubator. After that, the cells were fixed with ice-cold 4% PFA for 30 min and washed 3 times with PBS. Finally, specimens were fixed on slides using the mounting medium with DAPI (4’,6-diamino-2-phenolindole) that serves as a nuclear counterstain (FluoroshieldTM with DAPI, Sigma Aldrich, Munich, Germany). The specimens were observed using a confocal microscope (Leica TCS SPE, Leica Microsystems, KAWA.SKA Sp. z o.o., Zalesie Górne, Poland). The images were captured under × 630 magnification. Obtained photographs were merged using the ImageJ Software (version 1.52n, Wayne Rasband, National Institutes of Health, USA).

### Evaluation of proteins expression

The extracellular level of expressed proteins was established using Western Blot technique. Briefly, the cells from each experimental group were lysed using 100 µL of ice-cold RIPA Buffer supplemented with 1% addition of proteinase/phosphatase cocktail (Sigma Aldrich, Munich, Germany). The BCA method was used to determine the amount of isolated proteins (Bicinchoninic Acid Assay Kit, Sigma Aldrich, Munich, Germany). The samples containing 20 µg of protein were mixed with Laemmli loading buffer in the ration 4:1 respectively and incubated at 95 °C for 5 min in T100 Thermal Cycler (Bio-Rad, Hercules, CA, USA). SDS-PAGE was performed in Mini-PROTEAN Tetra Vertical Electrophoresis Cell (Bio-Rad, Hercules, CA, USA) using 10% sodium dodecyl sulphate–polyacrylamide gel for 90 min at 100 V. Afterwards, the gels were transferred into PVDF membranes using the Mini Trans-Blot® system (Bio-Rad, Hercules, CA, USA). The reaction last 60 min at 100 V. Then, the membranes were blocked in 5% skim milk powder in TBST for 60 min and incubated with primary antibodies overnight at 4 ˚C. The incubation with secondary antibodies was performed for 120 min at 4 ˚C. The list of used antibodies is attached in Table 1. Finally, the membranes were analysed using Bio-Rad ChemiDoc™ XRS system (Bio-Rad, Hercules, CA, USA) and DuoLuX® Chemiluminescent and Fluorescent Peroxidase (HRP) Substrate (Vector Laboratories). Image Lab™ Software (Bio-Rad, Hercules, CA, USA) was used to calculate the intensity and molecular weights of detected proteins.

**Table 1:** List of the antibodies used during this study

Detected protein	Host	Antibody dilution used in WB	Catalog no	Equine Cross-Reactivity	Manufacturer
**Primary antibodies**					
IRE-1	Human	1:1000	orb184380	+	Biorbyt
CHOP	Mouse	1:1000	2895 T	/	Cell Signalling Technology
eiF2a	Rabbit	1:1000	nbp2-67,353	+	Novus
PDIA3	Rabbit	2 µg/mL	arp63565	+	Aviva
INSR	Mouse	1 µg/mL	MA1-10,865	/	Invitrogen
IRS-1	Rabbit	1:1000	orb6236	/	Biorbyt
GLUT-4	Rabbit	1:1000	orb10728	/	Biorbyt
β-ACT	Mouse	1:2500	a5441	/	Sigma-Aldrich/Merck
**Secondary antibodies**					
Goat Anti-Rabbit IgG Antibody, Fc, HRP conjugate	/	1:2500	ap156p	/	Sigma-Aldrich/Merck
Anti-Mouse IgG (Fc specific)–Peroxidase antibody produced in goat	/	1:10,000	A0168	/	Sigma-Aldrich/Merck

### Evaluation of genes expression

The mRNA and miRNA expression were measured using RT-qPCR technique. The cells from each experimental group were homogenized using 1 mL of Extrazol® (Blirt DNA, Gdańsk, Poland). The RNA was isolated using well-established phenol–chloroform method. The quantity and purity of isolated total RNA was evaluated spectrophotometrically at 260 and 280 nm wavelength (Epoch, Biotek, Bad Friedrichshall, Germany). 500 ng of RNA was used for gDNA digestion. The reaction was carried out using DNase I, RNase-free Kit (Thermo Scientific, EN0525). Afterwards, the cDNA was synthesised using PrimeScript™ RT Master Mix (Takara, cat. no. RR036A) and Mir-X™ miRNA First-Strand Synthesis Kit (Takara Clontech Laboratories, Biokom, Poznań, Poland) in T100 Thermal Cycler (Bio-Rad, Hercules, CA, USA) accordingly to producers’ protocol. The qPCR reaction was performed in CFX Connected Real-Time PCR Detection System (Bio-Rad, Hercules, CA, USA) using SensiFAST SYBR®&Fluorescein Kit (Bioline Reagents Ltd., London, UK). The characterization of primers with annealing temperatures is attached in Table 2. The following cycling conditions were maintained in 10 µL volumes: 2 min of initial denaturation in 95 ˚C and 40 cycles of denaturation (15 s, 95 ˚C), annealing (15 s) and elongation (15 s, 72 ˚C). The values of transcripts expression were calculated using RQ MAX algorithm (log scale). The GAPDH and snU6 were used as reference transcripts.

**Table 2:** List of the genes used during this study

Gene	Primer	Sequence 5'–3'	Amplicon length (bp)	Accession No
*ATF6*	F: R:	CAGGGTGCACTAGAACAGGG AATGTGTCTCCCCTTCTGCG	164	XM_023640315.1
*p53*	F: R:	TTTCGACATAGCGTGGTGGT CTCAAAGCTGTTCCGTCCCA	252	NM_001202405.1
*p21*	F: R:	GAAGAGAAACCCCCAGCTCC TGACTGCATCAAACCCCACA	241	XM_023633878.1
*IRE-1*	F: R:	GAATCAGACGAGCACCCGAA TTTCTTGCAGAGGCCGAAGT	300	XM_023652216.1
*Bip*	F: R:	GTATGTCTTCGGCAACGGGA CAACTGACGTCACCGCTACT	122	XM_023628864.1
*eiF2a*	F: R:	GGTGAACGGACCACCACATT GGCGAGAACTCAAGGCAAAC	489	XM_001490053.5
*cebpa*	F: R:	CTGGAGCTGACCAGTGACAA GAGACCCTGAGACCCGAAAC	116	XM_023649498.1
*PPARγ*	F: R:	TCCCTGTTTGTGTACAGCCTT CTCCATGGCTGATTTCCCCT	191	XM_014846252.1
*SOD1*	F: R:	CATTCCATCATTGGCCGCAC GAGCGATCCCAATCACACCA	130	NW_001867397.1
*SOD2*	F: R:	GGACAAACCTGAGCCCCAAT TTGGACACCAGCCGATACAG	125	NW_001867408.1
*INSR*	F: R:	CAGTCAACGAGTCTGCCAGT CCCGGTGCACAAACTTCTTG	303	NM_001081866.2
*IRS-1*	F: R:	CTGCTGGGGGTTTGGAGAAT TAAATCCTCACTGGAGCGGC	254	XM_023642446.1
*GLUT-4*	F: R:	CGGGTTTTCAACAGATCGGC CACCTTCTGTGGGGCATTGA	658	XM_014862015.1
*GAPDH*	F: R:	GATGCCCCAATGTTTGTGA AAGCAGGGATGATGTTCTGG	250	NM_001163856.1
*CHOP*	F: R:	AGCCAAAATCAGAGCCGGAA GGGGTCAAGAGTGGTGAAGG	272	XM_001488999.4
*PERK*	F: R:	GTGACTGCAATGGACCAGGA TCACGTGCTCACGAGGATATT	283	XM_023618757.1
*CAT*	F: R:	ACCAAGGTTTGGCCTCACAA TTGGGTCAAAGGCCAACTGT	112	XM_014729341.2
*GPx*	F: R:	TCGAGCCCAACTTCACACTC AAGTTCCAGGCGACATCGTT	178	NM_001166479.1
*miR-7a-5p*	-	TGGAAGACTAGTGATTTTGTTGT	-	MIMAT0000677
*miR-21-5p*	-	TAGCTTATCAGACTGATGTTGA	-	MIMAT0000076
*miR-24-3p*	-	TACCACAGGGTAGAACCACGGA	-	MIMAT0000080
*miR-140-3p*	-	TACCACAGGGTAGAACCACGGA	-	MIMAT0012927

### Statistical analysis

Each analysis was performed in at least three technical repetitions and analysed using GraphPad Prism 8.0.2 (GraphPad Software, San Diego, CA, USA). The statistics were calculated using one-way analysis of variance (ANOVA) and Tukey’s Post-hoc test. The levels of significance were indicated with asterisks: * for *p* < *0.05*, ** for *p* < *0.01* and *** for *p* < *0.001*. The differences were considered as significant with * *p* < *0.05*.

## Results

### In vitro cytocompatibility of SHBG on EqASC_HE_ and EqASC_EMS_ cells

**Fig. 1:**
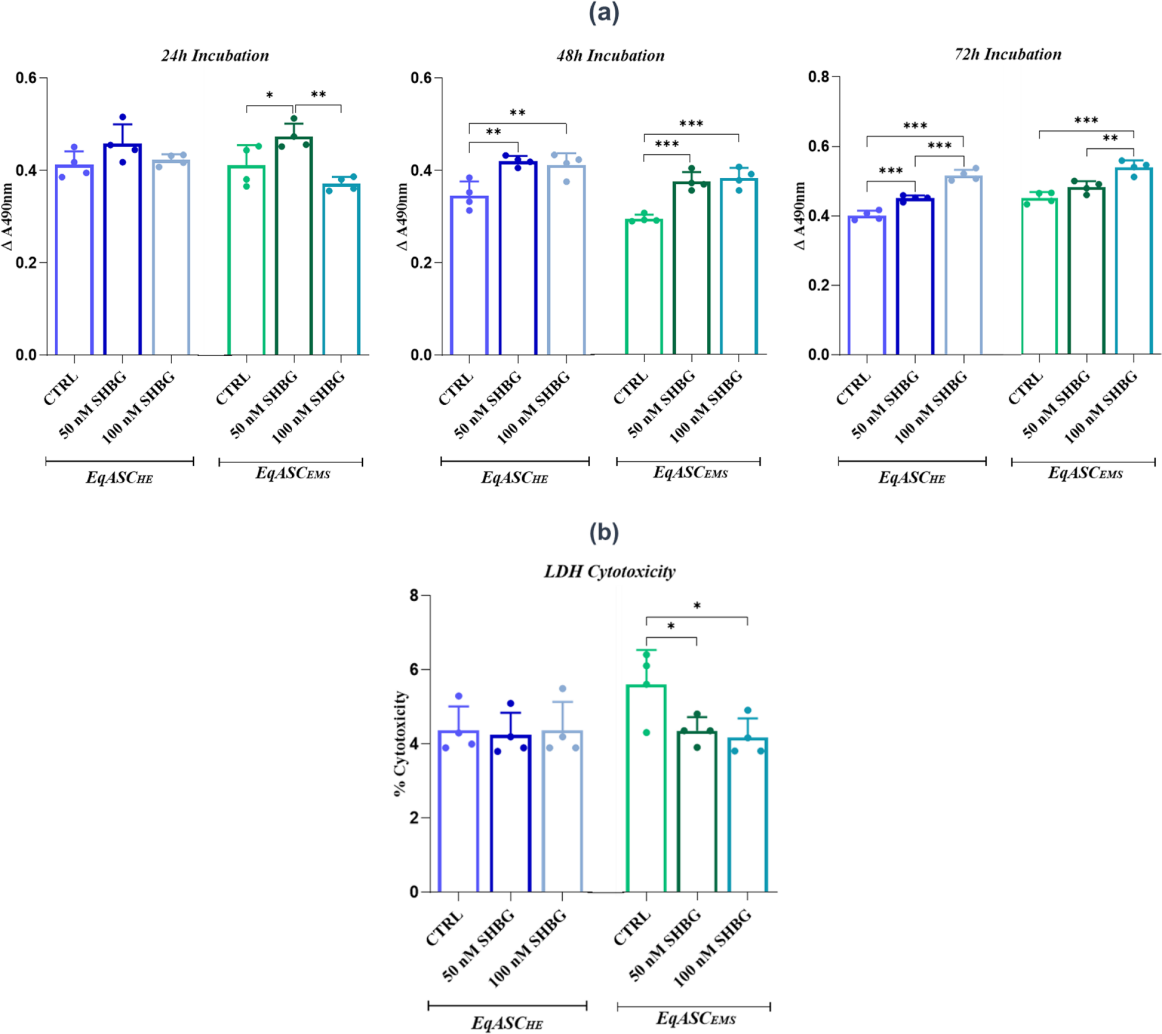
Effect on cell proliferation and viability of sex hormone binding globulin (SHBG) on EqASC_EMS_. **a** Histograms represent the average absorbance at 490 nm of MTS tetrazolium. **b** Histograms represent the average absorbance at 490 nm and 680 nM of lactate dehydrogenase (LDH). Representative data from three independent experiments are shown ± SD (*n* = 4). An asterisk (*) indicates a comparison of treated group to untreated healthy cells. * *p* < *0.05*, ** *p* < *0.01*, *** *p* < *0.001*. EqASC_HE_: healthy equine stem cells; EqASC_EMS_: EMS equine stem cells; EqASC_EMS_ 50 nM SHBG: equine EMS stem cells treated with 50 nM of SHBG for 24 h; EqASC_EMS_ 100 nM SHBG: equine EMS stem cells treated with 100 nM of SHBG for 24 h

In order to evaluate the effect of SHBG on the viability of ASC from healthy and EMS affected horse, MTS tetrazolium metabolization assay and ELISA-based lactate dehydrogenase (LDH) determination were performed. The cells were cultured in the presence of two different concentrations (50 and 100 nM) of the SHBG protein for 24, 48 and 72 h. The results obtained did not reveal any deleterious effect on the cell viability of EqASC_HE_ and EqASC_EMS_ 24, 48 and 72 h of incubation respectively (Fig. 1a). The LDH cytotoxicity test was carried out after 24 h of incubation with the SHBG, the result shows that the protein has no toxic effect on the cells (Fig. 1b).

### SHBG reduces apoptosis in EqASC_EMS_ cells

**Fig. 2:**
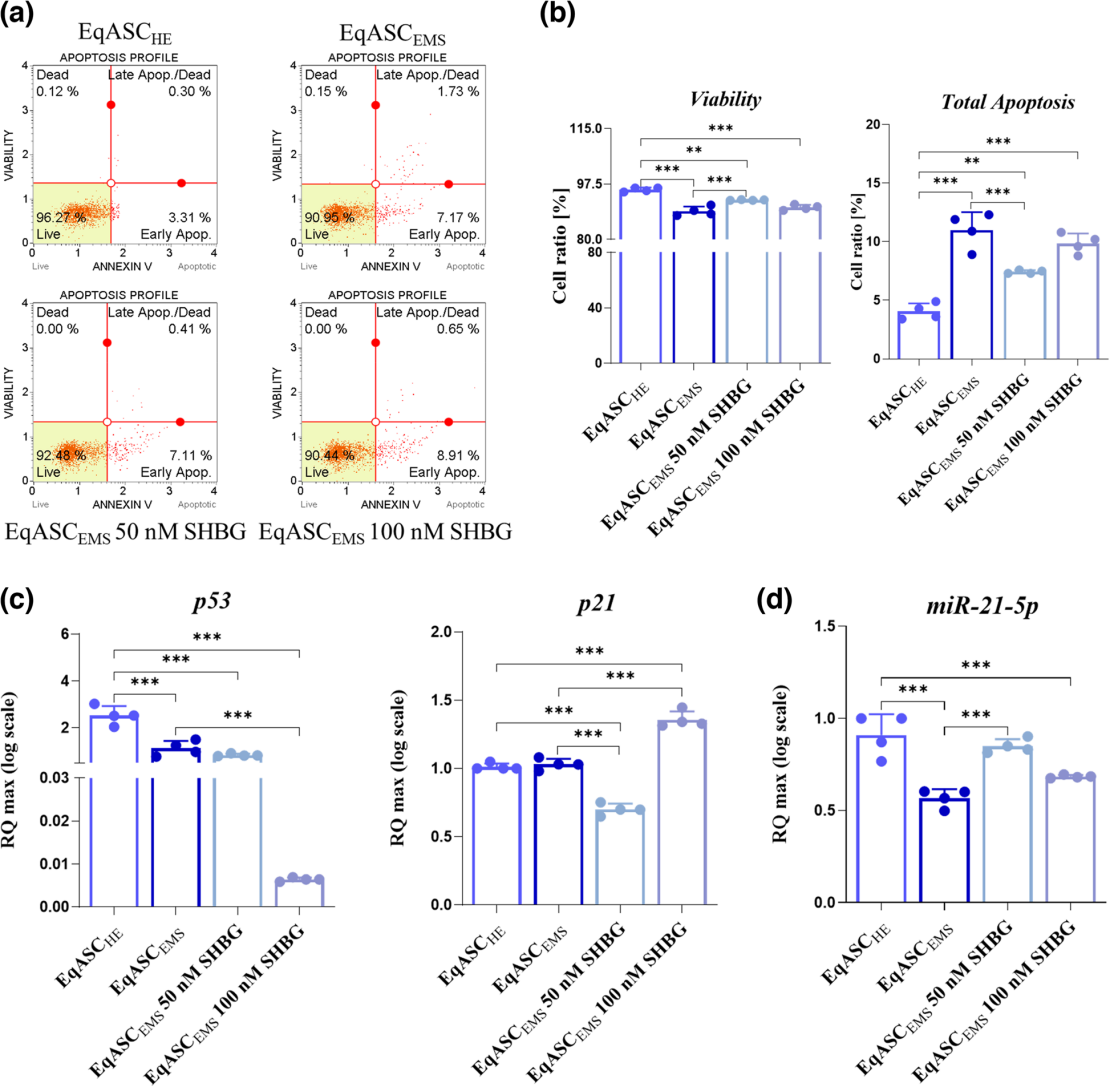
The influence of SHBG on the regulation of cells apoptosis in the EqASC_EMS_. **a** Representative dot-plots for Annexin V & Dead Cell assay. **b** Bar-charts depicting the quantitative analysis of live, early, and late apoptosis and cell death. **c** Relative expression quantitation of main apoptosis-associated markers levels (*p53* and *p21*). **d** Relative expression quantitation of apoptosis-associated *miR-21-5p* level. Representative data from three independent experiments are shown ± SD (*n* = 4). An asterisk (*) indicates a comparison of treated group to untreated healthy cells. * *p* < *0.05*, ** *p* < 0.01, *** *p* < 0.001. EqASC_HE_: healthy equine stem cells; EqASC_EMS_: EMS equine stem cells; EqASC_EMS_ 50 nM SHBG: equine EMS stem cells treated with 50 nM of SHBG for 24 h; EqASC_EMS_ 100 nM SHBG: equine EMS stem cells treated with 100 nM of SHBG for 24 h

In order to examine whether the exogenous SHBG treatment affects the EqASC_EMS_-associated.

apoptosis, the latter were analysed using Muse Annexin V & Dead Cells assay, the gene expression of markers linked to apoptosis (*p53* and *p21*) and miRNAs involved mainly in the modulation of the apoptosis (*miR-21-5p*) were examined using RT-qPCR respectively (Fig. 2). In addition, the results obtained during the flow cytometry analysis for the Annexin V & Dead Cell on the rate of living and dead cells show that the cell survival rate is reduced in the EqASC_EMS_ with an average of 90% of living cells compared to the control group EqASC_HE_ with an average > 95% of living cells; furthermore, cell survival rate is upregulated in SHBG treated groups (> 92%) compared to EMS cells (< 90%), demonstrating the cell proliferation-enhancing effect of SHBG with a better action when the concentration of the latter is 50 nM (> 92%) compared to 100 nM (> 90%) (Fig. 2a/2b). As shown in Fig. 2c, the results obtained from the RT-qPCR analysis of the apoptotic markers cited above demonstrate that the expression of *p53* was significantly decreased in the EqASC_EMS_ cell group in contrast to the healthy EqASC_HE_ control group (*p* < *0.001*) and the expression of *p21* is slightly increased in the EqASC_EMS_ group. After treatment of EMS cells with the two concentrations of SHBG (50 and 100 nM) a significant decrease was observed for the *p53* and *p21* genes expression *(p* < *0.001)* with a better effect when the concentration of SHBG is 50 nM. In addition, miRNAs involved in apoptosis were analysed using RT-qPCR (Fig. 2d), the obtained results show a lower expression of *miR-21-5p* in EqASC_EMS_ compared to the healthy control group EqASC_HE_ *(p* < *0.001)*. However, after administration of the exogenous SHBG treatment (50 and 10 nM), it was observed that the expression of the miRNAs *miR-21-5p* appears to be up-regulated in both groups (EqASC_EMS_ 50 nM SHBG and EqASC_EMS_ 100 nM SHBG); however, it appears that the treatment is more effective when the dose of exogenous SHBG is 50 nM.

### SHBG enhances EqASC_EMS_’ cell cycle

**Fig. 3:**
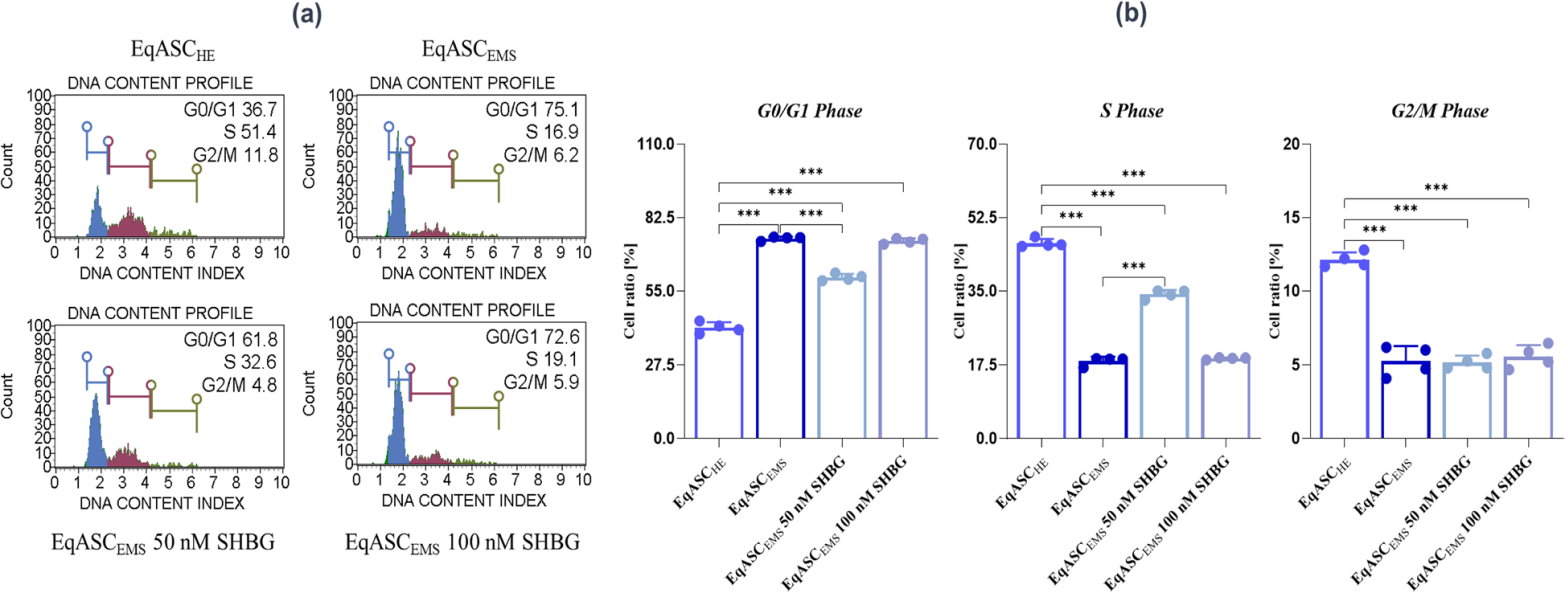
The effect of the SHBG on EqASC_EMS_’ cell cycle. **a** Representative dot-plots for cell cycle analysis of DNA content by the Muse™ Cell Cycle assay. **b** Bar-charts depicting the quantitative analysis of G0/G1, S and G2/M phases. Representative data from three independent experiments are shown ± SD (*n* = 4). An asterisk (*) indicates a comparison of treated group to untreated healthy cells. * *p* < *0.05*, ** *p* < *0.01*, *** *p* < *0.001*. EqASC_HE_: healthy equine stem cells; EqASC_EMS_: EMS equine stem cells; EqASC_EMS_ 50 nM SHBG: equine EMS stem cells treated with 50 nM of SHBG for 24 h; EqASC_EMS_ 100 nM SHBG: equine EMS stem cells treated with 100 nM of SHBG for 24 h

The determination of the impact of the SHBG protein on the cell cycle was carried out by measuring the DNA content of cells at different stages of the cell cycle which were evaluated and determined using the Muse™ Cell Cycle test (Fig. 3a/b). Cells affected by EMS (EqASC_EMS_) show a marked increase in the cell population of the G0/G1 phase compared to the healthy control group (EqASC_HE_) *(p* < *0.001)*, which indicates that the EMS cells are in a state of cell death. After administration of the SHBG protein at a rate of 50 and 10 nM, a significant drop *(p* < *0.01)* in the percentage of the G0/G1 cell population is observed in the EqASC_EMS_ 50 nM SHBG cells, on the other hand there is no remarkable difference when the dose of SHBG is at 100 nM (EqASC_EMS_ 100 nM) compared to the control EMS group (EqASC_EMS_). In addition, the levels of the cell population of the S phase are significantly under regulated in the EMS cells compared to the healthy control group (*p* < *0.001*), but become significantly regulated in the groups treated with SHBG with a statistically significant better action when the concentration of the latter is 50 nM *(p* < *0.001)*. No significant changes in cell populations were identified in the G2/M phase of the cell cycle of EMS cells and SHBG treated cells groups.

### SHBG mitigates endoplasmic reticulum stress in EqASC_EMS_ cells

**Fig. 4:**
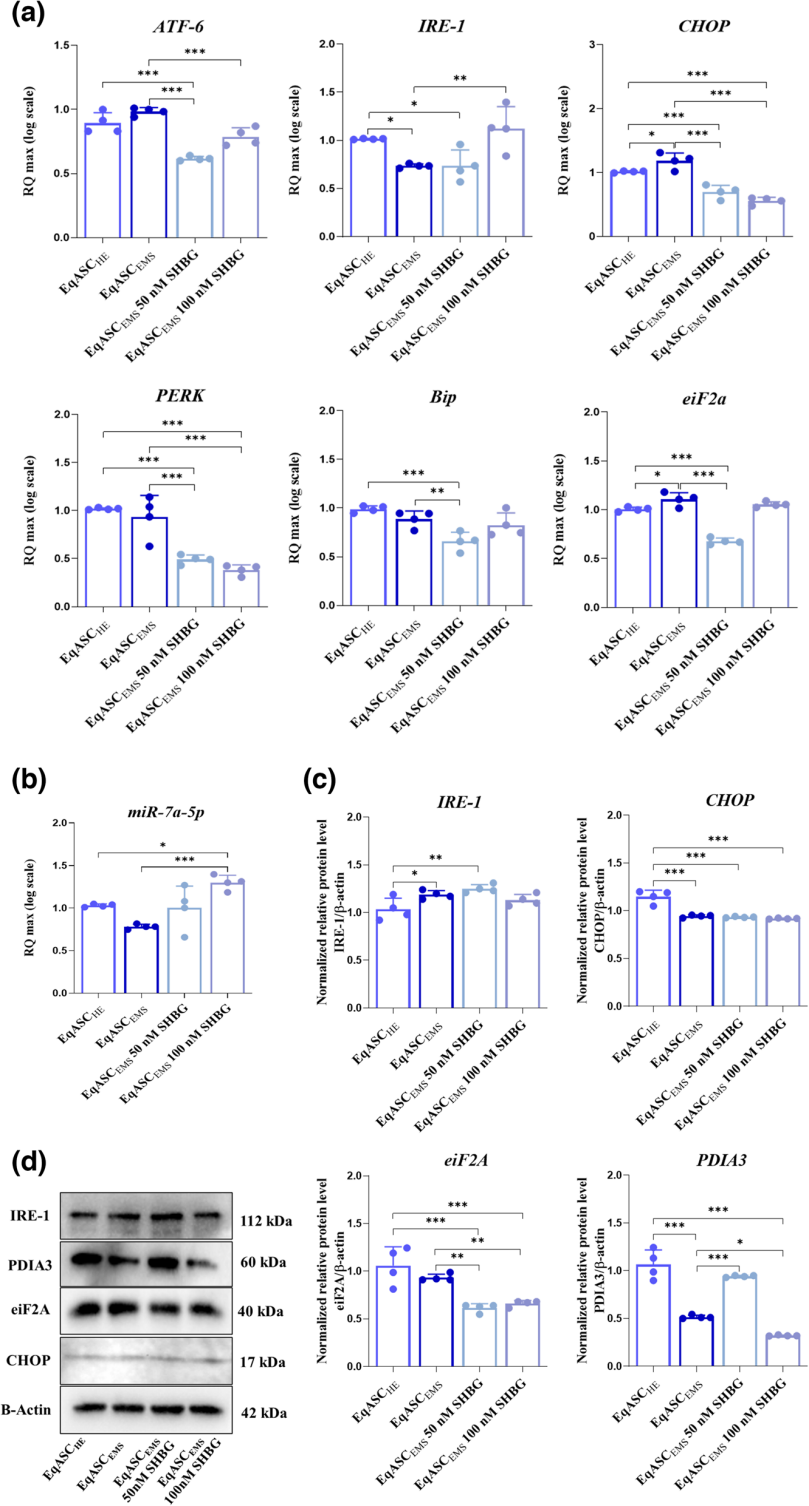
Preventive effect of SHBG in EqASC_EMS_ cells against endoplasmic reticulum stress. **a** Relative gene expression of *ATF6*, *IRE1*, *CHOP*, *PERK*, *Bip* and *eiF2A* ER stress transcripts. **b** Relative expression quantitation of ER stress-associated *miR-7a-5p* level. **c** Quantitative representation of IRE1, CHOP, eiF2A and PDIA3 proteins. **d** Representative immunoblots for each assayed protein detected by chemiluminescence. Representative data from three independent experiments are shown ± SD (*n* = 4). An asterisk (*) indicates a comparison of treated group to untreated healthy cells. * *p* < *0.05*, ** *p* < *0.01*, *** *p* < *0.001*. EqASC_HE_: healthy equine stem cells; EqASC_EMS_: EMS equine stem cells; EqASC_EMS_ 50 nM SHBG: equine EMS stem cells treated with 50 nM of SHBG for 24 h; EqASC_EMS_ 100 nM SHBG: equine EMS stem cells treated with 100 nM of SHBG for 24 h

In order to elucidate whether the sex hormone binding globulin manages to regulate endoplasmic reticulum stress in EqASC_EMS_, the expression of the *ATF6*, *IRE-1*, *CHOP*, *PERK*, *Bip*, *eiF2A* genes and *miR-7a-5p*, as well as expression of the IRE-1, CHOP, eiF2A and PDI3 proteins which are considered to be the main regulators of ER stress was assessed using RT-qPCR and western blot respectively (Fig. 4). As showed in the obtained results from the RT-qPCR, the EqASC_EMS_ were characterized by marked overexpression of *ATF6*, *CHOP* and *eiF2A* transcripts by contrast to the healthy control group EqASC_HE_ (*p* < *0.05*), which underlines the establishment of an ER stress upon EMS onset. After treatment of the cells with the two different concentrations of SHBG (50 and 100 nM), a slight decrease in the expression levels of above cited genes was observed; what is more, it appeared that the 50 nM concentration was more efficient for the decrease of both *ATF-6* and *eiF2A* genes as compared to the highest concentration (100 nM) concentration (Fig. 4a). However, we also note that the genetic markers *IRE1*, *PERK* and *Bip* are not upregulated in the control EMS cells (EqASC_EMS_) compared to the group of healthy control cells (EqASC_HE_) (Fig. 4a). Concerning the expression of *miR-7a-5p*, it appears that its expression is downregulated when the ASCs cells undergo an EMS state (Fig. 4b); in contrast, the exogenous SHBG treatment upregulated its expression in both EqASC_EMS_ 50 nM SHBG and EqASC_EMS_ 100 nM SHBG (Fig. 4b). The analysis of protein expression revealed that the expression of PDIA3, CHOP and eiF2A proteins were significantly under regulated in EMS cells compared to healthy cells (*p* < *0.001*) (Fig. 4c/d), and in parallel, protein expression of IRE-1 was significantly higher than normal in EqASC_EMS_ (*p* < *0.01*) (Fig. 4c/4d), indicating a state of ER stress; however, after treatment of the cells with SHBG, it can be observed that the protein level of IRE-1 remains high compared to the healthy control group (EqASC_HE_) (*p* < *0.01; p* < *0.001*) and that the protein expression of PDIA3 was significantly upregulated *(p* < *0.001)* for the EqASC_EMS_ 50 nM SHBG; at the same time, the expression of CHOP and eiF2A was not variating after the exogenous SHBG treatment (Fig. 4c/d).

### SHBG attenuates oxidative stress in EqASC_EMS_ cells

**Fig. 5:**
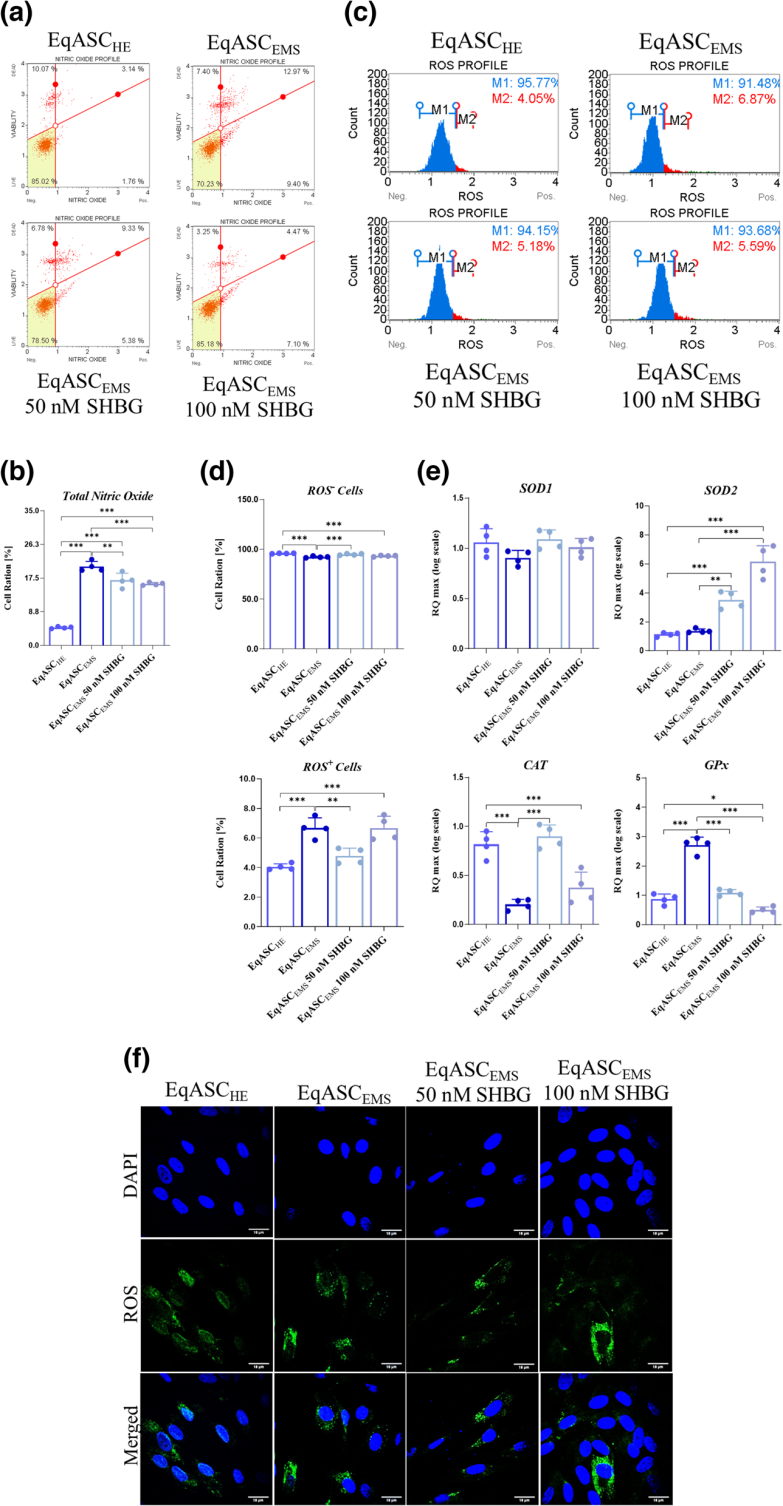
Sex hormone binding globulin (SHBG) moderates oxidative stress in EqASC_EMS_. **a** Dot-Plots for intracellular nitric oxide production detected by Muse**™** Nitric Oxide Kit. **b** Average percentages of total nitric oxide production cells in each experimental group. **c** Dot-Plots for intracellular ROS production detected by Muse™ Oxidative Stress Kit. **d** Average percentages of total ROS^+^ and ROS^−^ cells in each experimental group. **e** Relative gene expression of *Sod1*, *Sod2*, *CAT* and *GPx* antioxidant enzymes transcripts. **f** Representative photomicrographs of CM-H2DCFDA staining assay obtained by confocal epi-fluorescent microscopy; Bar size 18 μm; magnification × 60. Representative data from three independent experiments are shown ± SD (*n* = 4). An asterisk (*) indicates a comparison of treated group to untreated healthy cells. * *p* < *0.05*, ** *p* < *0.01*, *** *p* < *0.001*. EqASC_HE_: healthy equine stem cells; EqASC_EMS_: EMS equine stem cells; EqASC_EMS_ 50 nM SHBG: equine EMS stem cells treated with 50 nM of SHBG for 24 h; EqASC_EMS_ 100 nM SHBG: equine EMS stem cells treated with 100 nM of SHBG for 24 h

To examine the potential activity of SHBG on oxidative stress in EMS cells, the percentage of ROS positive and negative cells was quantified using the Muse™ Oxidative Stress Kit, nitric oxide (NO) levels were determined using the Muse**™** Nitric Oxide Kit, gene expression of oxidative stress-related markers was analysed using RT-qPCR and additionally, confocal epi-fluorescent microscopy was performed using the CM-H2DCFDA staining reagent was made (Fig. 5). The results obtained show a significant increase in the production of nitric oxide in EMS cells compared to the healthy control cells *(p* < *0.001)*; but after treatment with the 2 concentrations of SHBG (50 and 100 nM), nitric oxide levels appeared to be significantly down-regulated *(p* < *0.01; p* < *0.001)*. Moreover, the results obtained during the analysis of the rate of ROS^+^ cells (Fig. 5c/d), an increase in the percentage of ROS^+^ cells is observed in cells affected by EMS (> 6%) compared to the control healthy cells group (± 4%) *(p* < *0.01)*. This demonstrates that EMS cells exhibit a significant accumulation of intracellular reactive oxygen species (ROS), which implies the occurrence of oxidative stress within the affected cells. Treatment with SHBG excreted an antioxidant effect, as indicated by the large decrease in the total number of ROS^+^ cells *(p* < *0.001)*, especially in the EqASC_EMS_ 50 nM SHBG group. Moreover, gene expression analysis of the four main endogenous antioxidant enzymes, namely *Sod1*, *sod2*, *Cat* and *GPx*, revealed that the mRNA levels of *Sod1* were strongly decreased, while those of *Sod2*, *Cat* and *GPx* were significantly upregulated in response to ROS overproduction under EMS conditions (Fig. 5c). However, the administration of SHBG effectively regulated the expression patterns of *Sod1*, *Cat* and *GPx* which reveals the antioxidant effect of the SHBG protein on EqASC_EMS_.

### SHBG ameliorates insulin signalling pathway in EqASC_EMS_ cells

**Fig. 6:**
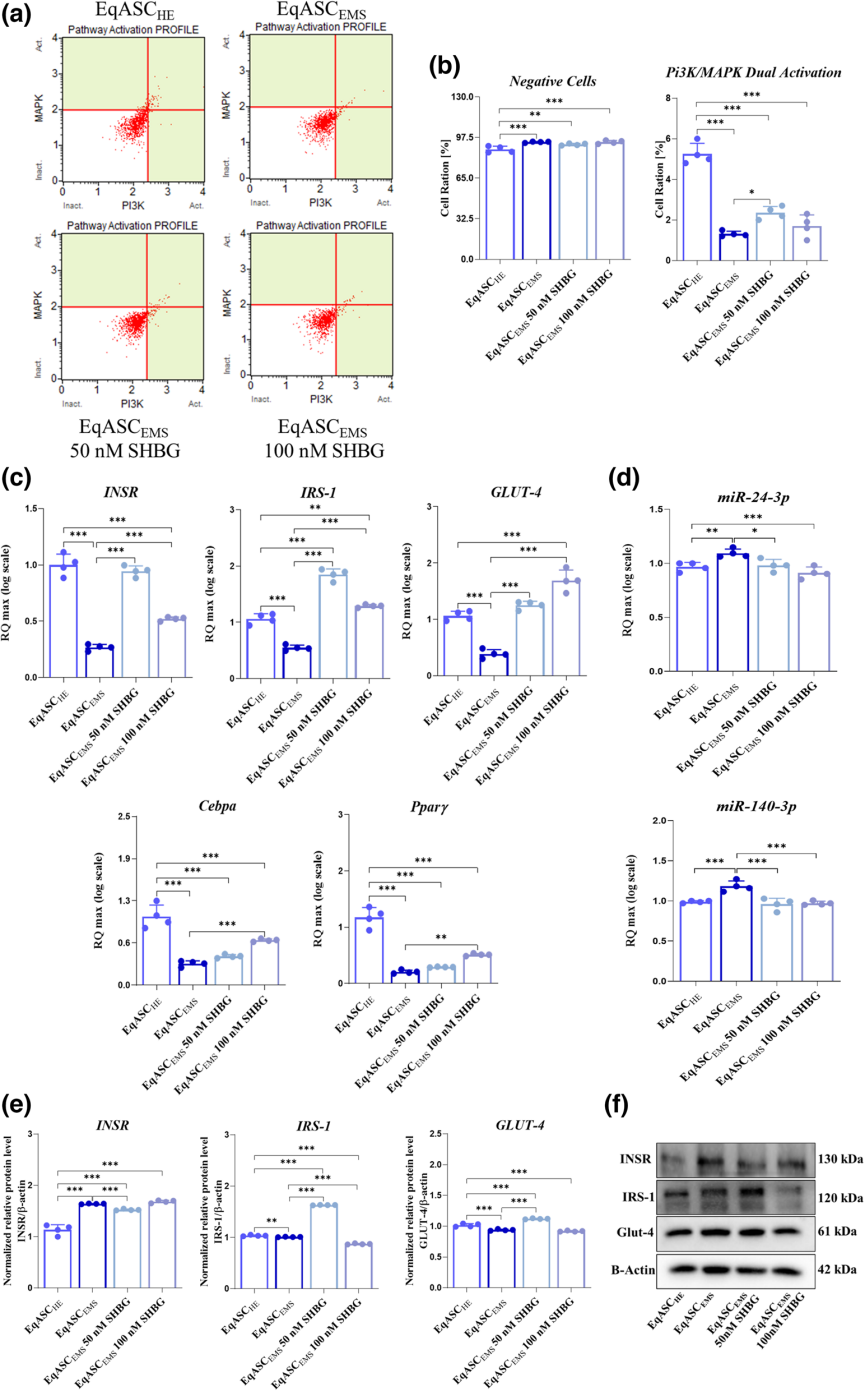
Reversing effect of sex hormone binding globulin on defective insulin signalling in EqASC_EMS_. **a** Dot-Plots for the dual Pi3K/MAPK pathway activation. **b** Average percentages of negative and positive Pi3K/MAPK pathway activation in each experimental group. **c** Main genes (*INSR*, *IRS-1*, *Glut-4*) expression levels involved in insulin signalling execution. **d** Relative expression quantitation of insulin signalling pathway *miR-24-3p* and *miR-140-3p* levels. **e** Quantitative analysis of insulin signalling-related proteins expression using western blot. **f** Representative immunoblots for protein insulin signalling detected by chemiluminescence. Representative data from three independent experiments are shown ± SD (*n* = 4). An asterisk (*) indicates a comparison of treated group to untreated healthy cells. * *p* < *0.05*, ** *p* < *0.01*, *** *p* < *0.001*. EqASC_HE_: healthy equine stem cells; EqASC_EMS_: EMS equine stem cells; EqASC_EMS_ 50 nM SHBG: equine EMS stem cells treated with 50 nM of SHBG for 24 h; EqASC_EMS_ 100 nM SHBG: equine EMS stem cells treated with 100 nM of SHBG for 24 h

Insofar insulin resistance constitutes on one the most prominent hallmarks of EMS, the potential of SHBG treatment to restore insulin signalling in EMS-affected ASC has been further investigated. As shown in the Fig. 6a/b, EqASC_EMS_ cells were characterized by defective insulin-stimulated Pi3K/MAPK pathway activation, as evidenced by the decrease in the percentage of cells exhibiting dual phosphorylated Akt(Ser473) and ERK1/2 (Thr202/Tyr204, Thr185/Tyr187), when compared to healthy ASC cells (EqASC_HE_) (*p* < *0.001*). Application of SHBG at 50 nM to the EMS cells resulted in an enhancement of the Pi3K/MAPK axis activation under insulin stimulation, characterized by the augmentation of the proportion of ERK1/2-Akt activated cells, which refers to the dual MAPK and PI3K pathway activation and underlying restoration of Akt and ERK phosphorylation events (*p* < *0.05*). Nevertheless, highest concentration of SHBG did not increase the percentage of cells with activated Pi3K/MAPK axis compared to EMS untreated cells (Fig. 6a/b). To further confirm the positive effect of SHBG glycoprotein on insulin signal transduction, protein, gene and miRNA expression of master transducers has been analysed. As depicted in the Fig. 6c/d/e/f, EMS-derived ASC cells exhibited impaired insulin signalling as observed through the significant downregulation of *INSR*, *IRS-1* and *Glut-4* transcripts by opposition to control group (*p* < *0.001*). Moreover, as shown in the Fig. 6d the expression of *miR-24-3p* and *miR-140-3p* were significantly overexpressed in the EqASC_EMS_ affected cells compared to the healthy control group EqASC_HE_ *(p* < *0.001).* The protein expression patters of the same genes markers similarly appeared dysregulated in EMS untreated cells, where INSR expression was increased while those of IRS-1 and Glut-4 were found to be reduced (*p* < *0.05*; *p* < *0.001*). Interestingly, the incubation of EMS ASC cells with SHBG protein resulted in an obvious enhancement of insulin-related mediators’ expression. Indeed, the expression of *INSR*, *IRS-1* and *Glut-4* was increased at mRNA level following the application of SHBG at both concentrations (50 and 100 nM) when compared to EMS untreated group of cells (*p* < *0.001*). The expression of *miR-24-3p* and *miR-140-3p* were also downregulated in the SHBG-treated group. Surprisingly, SHBG at a concentration of 50 nM restored the protein levels of both IRS-1 and Glut-4 compared to EMS control group (*p* < *0.001*), while highest concentration did not induce any significant improvement in the protein expression of the studied factors (Fig. 6d/e). The expression of INSR for its part was found to be reduced upon SHBG supplementation at a concentration of 50 nM, and inversely augmented at the highest SHBG concentration, i.e., 100 nM.

## Discussion

Equine metabolic syndrome (EMS) is a collection of multiple clinical factors that include, systemic inflammation, liver lipotoxicity, general insulin resistance (IR), and finally excessive accumulation of pro-inflammatory cytokines, oxidative stress mediators, and metalloproteinases in adipose tissue. The resulting tissue distress causes significant alteration of residing adipose-derived mesenchymal stem cells (ASC) in terms of viability, proliferation, expansion, multilineage differentiation potential and metabolic functions [4]. SHBG is a hepatokine involved in sex hormones bioavailability regulation, however, recent lines of evidence suggested that it may also play important role in the mediation of various other metabolic cellular processes. Indeed, we previously demonstrated that SHBG is present and expressed not only in liver but also in adipocytes and their progenitors; which levels are seriously diminished in EMS mares which correlates with adipocytes and ASCs molecular and functional dysfunctions [22]. In the presented study, we have found that SHBG mitigates ER stress and improves cells viability and insulin sensitivity in adipose-derived mesenchymal stem cells (ASC) isolated from metabolic syndrome-affected horses (EMS).

Molecular EMS bias have been demonstrated to encompass increased pro-apoptotic signals in adipose tissue, as a result of the characteristic metabolic overload and accumulation of reactive intermediates that trigger general cellular repair mechanisms failure [23]. In our investigation, we found that ASC derived from EMS suffering horses displayed critical reduced cellular viability, proliferation capacity and resulting augmented apoptosis. Moreover, we observed that treatment of affected cells with exogenous SHBG significantly improved the metabolic activity of EqASC_EMS_ by promoting their proliferation rate and cell cycle. Moreover, SHBG application reversed ASC_EMS_ apoptosis by reducing the expression of *p53* and *p21* mRNA and increasing cell survival rate at 50 nM. Currently, there are no reports showing the pro-survival effect of SHBG protein on non-cancerous cells; however, previous findings evidenced the necessity of a C terminus SHBG-like domain for efficient neuronal protective effect of the anticoagulant factor protein S (PS) under hypoxic milieu. Indeed, the study demonstrated that the ability of the PS protein to increase the levels of Bad, Bcl-2 and Bcl-XL pro-survival factors and reduce the levels of proapoptotic proteins p53 and Bax was strictly mediated by its SHBG-domain [24]. The exact mechanisms by which SHBG may promote cell survival and abrogate apoptosis are not yet elucidated, however, in the present study, we showed that SHBG application increased the activation of the MAPK pathway, and thus stimulated the phosphorylation of ERK1/2 mediators.

Transient or prolonged activation of ERK1/2 has been found to prevent programmed cell death and to exert anti-apoptotic activity under various stress stimuli including inflammation and oxidative stress [25]. Another possible molecular target underlying SHBG pro-survival effect can be represented by the protein disulfide‑isomerase A3 (PDIA3) thiol oxidoreductase. Our data demonstrated that EMS ASC exhibited suppressed PDI3A protein expression, which has been significantly restored following exogenous SHBG treatment. PDIA3 has in fact been reported in several studies as a potent chaperone that protects cells from apoptotic pathways. By limiting the accumulation of misfolded proteins, PDI superfamily members act as limiting factors for ER stress response and resulting apoptosis associated to oxidative stress, to promote cellular survival and proliferation, which could explain our presented findings [26]. Taken together, these data suggest the great potential of SHBG as an anti-apoptotic and pro-survival protein in the course of EMS, that may act on the ERK/p53/Bcl-2 axis and the ER-associated PDIA3 chaperone.

Oxidative stress is recognized as a critical component of EMS [27], which occurs as a result of persistent low-grade inflammation, altered lipids and glucose metabolism and consequent mitochondrial dysfunction [28–31]. Therewith, earlier studies evidenced that under prooxidant milieu, ASC are losing their metabolic homeostasis and become more prone to apoptosis and ER stress, which substantially alter their regenerative properties [32]. Therefore, we were interested in verifying whether SHBG protein may mitigate oxidative stress and promote anti-oxidative defenses of EMS ASC cells. We found that SHBG reduced the levels of reactive oxygen species (ROS) and nitric oxide (NO) and at the same time enhanced the expression of total endogenous antioxidant enzymes including superoxide dismutase (SOD1/SOD2), catalase (CAT) and glutathione peroxidase (GPx), whose transcripts levels were dysregulated under EMS condition. Antioxidant effect of SHBG has not been tested previously on cellular or in vivo experimental models, however, our results are in agreement with other investigations that reported the close interrelation between low SHBG levels and oxidative stress severity. Sun and colleagues [33], demonstrated that insulin resistant patients characterized by decreased SHBG levels exhibited intensified oxidative stress evidenced by an elevation in circulating levels of both malondialdehyde (MDA) and oxidized low-density lipoprotein (ox-LDL), concomitantly to impaired antioxidant properties of high-density lipoprotein (HDL). Enli et *al.*, [34] similarly reported that SHBG loss is accompanied by lower levels of antioxidant enzymes and glutathione (GSH) under insulin resistance, hyperglycemia and dyslipidemia conditions. Additionally, intensified ROS, NO and nitrotyrosine production as well as xantine oxidase activity has been further noted in insulin resistant women lacking SHBG protein [35]—all of which corroborate the potential implication of SHBG in regulating and attenuation IR-associated oxidative stress.

Endoplasmic reticulum (ER) stress is recognized as a major risk factor for insulin resistance. EMS associated metabolic disturbances including hyperglycemia and hyperlipidemia are known to alter proteostasis and trigger the accumulation of unfolded or misfolded proteins, which together with increased ROS levels contribute to the initiation of the unfolded protein response (UPR) and related ER stress [36]. Due to profound metabolic failure, ASC derived from EMS horses display substantial ER stress and increased expression levels of both UPR sensors and effectors [23]. Here, we found that SHBG reduced the expression of PERK, eIF2α, Bip, CHOP and ATF6 in EqASC_EMS_. This observed effect is consistent with the results of our previous study, where we demonstrated that SHBG downregulated the expression of IRE1α, CHOP and ATF6 at both mRNA and protein levels in liver, highlighting its inhibitory effect toward UPR arms activation [37]. Under metabolic syndrome condition, accumulation of unfolded or misfolded proteins in the ER lumen induce the Bip protein to dissociate and bind to these proteins and subsequently activates the ER transmembrane sensors such as IRE1α*,* PERK/EIF2α and ATF6 through UPR signaling cascade, contributing to cells apoptosis and ER stress by ultimately initiate CHOP (ER stress-related apoptosis) [38, 39]. Hence, these findings evoke a regulatory effect of SHBG on UPR and ER stress events, and may represent an additional pro-survival and anti-apoptotic mechanism. Interestingly, SHBG similarly restored the expression of PDIA3 protein in EMS ASC. We above postulated that SHBG protein protected cells from programmed death via the stimulation of PDIA3 pro-survival protein expression. Similarly, this observed stimulation may also participate in the observed ER stress mitigation. As a matter of fact, besides its apoptosis inhibitory effects, PDIA3 is also a critical chaperone protein that participates in the glycoprotein-specific quality control machinery in ER lumen by regulating the expression of UPR effectors in order to attenuate the deleterious effects of cell stress such as ROS damage [40]. However, under sustained distress, PDIA3 expression is altered, and its deficiency has been found to result is an upregulation of GRP78, p-PERK, and CHOP, which further exacerbate ER stress responses [41]. Therefore, it can be postulated that SHBG exerts a retrograde effect on ER stress by at least inducing the expression of its PDIA3 chaperone.

Persistent hyperinsulinemia, impaired insulin sensitivity and disrupted glucose metabolism are recognized as a key components of EMS [42]. The loss in tissue responsiveness to insulin derive from a loss in intracellular signaling cascades activation for energy and lipid metabolism regulation, including insulin receptor (INSR), insulin receptor substrate (IRS) proteins, phosphoinositol 3-kinase/protein kinase B (Pi3K/Akt) as well as glucose transporter 4 (GLUT4) [43]. Here, we showed that exogenous SHBG application improved the overall expression of insulin signaling related transducers in ASC_EMS_. The crosstalk between SHBG availability and insulin transduction signal efficiency has previously been reported. Lowered expression levels of SHBG were associated with significantly decreased IRS-1, Pi3K/Akt and GULT4 mRNA and protein expression levels, which contributed to the establishment of local and systemic IR in human subjects [44]. When activated by INSR/Insulin complex, IRS1 tyrosine phosphorylation recruits the SH‐2 transforming proteins to phosphorylate phosphatidylinositol-3-kinase (Pi3K) to a phosphatidylinositol (3,4,5)-trisphosphate (PIP3) [45], which binds to phosphorylated PKB/Akt via phosphatidylinositol-dependent protein kinase-1 (PDK1) at the plasma membrane and further initiate GLUT4 translocation to the cell membrane [46]. Our data demonstrated that SHBG increased the phosphorylation levels of Akt at Ser473, as evidenced by the increased Pi3K/MAPK activation pathway, and upregulated the expression of C/EBPα and PPARγ, which have been shown as crucial mediators of insulin/Akt-stimulated GLUT4 glucose uptake in adipose progenitor cells [47], indicating the restoration of the signal transduction between Pi3K and Akt kinases. As a consequence, our findings further enlighten an upregulation of the GLUT4 transporter in EMS ASC cells treated with SHBG, pointing out the ability of SHBG to improve and restore insulin-responsive glucose absorption. These observations are corroborated with previous investigation, in which excess SHBG protein has been reported to increase the expression levels of GLUT1 cAMP/PKA/CREB1 pathway in insulin resistant trophoblasts [22], confirming that SHBG may improve glucose uptake in various cell types including ASC. Our study otherwise revealed an increase in INSR protein levels in EMS cells, while its gene expression appeared to be downregulated. Moreover, treatment with SHBG exerted a regulatory effect of INSR by promoting its gene expression and normalizing its protein level. Hyperinsulinemia, a salient EMS is known to trigger IR by dampening INSR autophosphorylation events, leading to a short-circuit in downstream signal transmission. Our observed data correlate with earlier research that reported a decrease in INSR tyrosine kinase activity and an elevation in total INSR protein level in a model of high-fat-diet induced hyperinsulinemia. Furthermore, it has been established that due to the non-responsiveness of INSR to insulin, INSR tend to accumulate at higher amounts in both cytosol and membrane and does not undergo typical degradation and recycling following its binding to insulin [48, 49]—all of which, substantiate the potent ability of SHBG to restore insulin downstream signaling and sensitivity.

## Conclusion

The present study confirmed that SHBG has improves equine EMS ASC viability, metabolic activity and protects against apoptosis and oxidative stress. SHBG through the recovery of PDIA3 ER chaperone protein expression, further mitigates ER stress as a part of its pro-survival potential. Furthermore, through the modulation of insulin signaling cascades, SHBG provides evidence of its insulin sensitizing effects on EqASC_EMS_ by potentiating the ISR-1/Pi3K/Akt/GLUT4 axis. These finds deliver new insights in using SHBG protein as a novel therapeutic target for metabolic syndrome and insulin resistance intervention.

### Supplementary Information

**Additional file 1: Figure 1.** Full-length blot for IRE-1 protein. **Figure 2.** Full-length blot for PDIA3 protein. **Figure 3.** Full-length blot for eiF2A protein. **Figure 4.** Full-length blot for CHOP protein. **Figure 5.** Full-length blot for β-Actin protein.

**Additional file 2: Figure 1.** Full-length blot for INSR protein. **Figure 2.** Full-length blot for IRS-1 protein. **Figure 3.** Full-length blot for Glut-4 protein. **Figure 4.** Full-length blot for β-Actin protein.

## Data Availability

All datasets generated and/or analysed during the current study are presented in the article, the accompanying Source Data or Supplementary Information files, or are available from the corresponding author upon reasonable request.

## Abbreviations

EMS: Equine Metabolic Syndrome
SHBG: Sex Hormone Binding Globulin
EqASCs_EMS_: EMS Affected Adipose-Derived Stromal Stem Cells
ER: Endoplasmic Reticulum
Pi3K: Phosphoinositide 3-kinase
MAPK: Mitogen-activated Protein Kinase
p53: Tumor Protein P53
p21: Cyclin-dependent Kinase Inhibitor 1
ROS: Reactive Oxygen Species
RNS: Reactive Nitrogen Species
PDIA3: Protein Disulfide-isomerase A3
Akt: Protein Kinase B (PKB)
Glut4: Glucose Transporter Type 4
ASCs: Adipose Tissue Derived Stem Cells
DNA: Deoxyribonucleic Acide
UPR: Unfolded Protein Response
CD90^+^: Cluster of Differentiation 90 (Thy-1) Positive Cells
CD105^+^: Cluster of Differentiation 105(Endoglin) Positive Cells
CD44^+^: Cluster of Differentiation 44 (Homing Cell Adhesion Molecule (HCAM)) Positive Cells
CD45-: Cluster of Differentiation 45 Negative Cells
Oct4: Octamer Binding Transcription Factor-4
SOX-2: Sex Determining Region Y Box-2
Nanog: Nanog Homeobox Protein
VEGF: Vascular Endothelial Growth Factor
BMP-2: Bone Morphogenic Protein
FGF: Fibroblast Growth Factor
Tregs: Regulatory Lymphocytes
SBP: Sex Steroid-binding Protein
KDa: Kilodalton
cAMP: Cyclic Adenosine Monophosphate
T2DM: Type 2 Diabetes
MetS: Metabolic Syndrome
IRE1α: Inositol Requiring Enzyme 1
CHOP: DNA Damage-inducible Transcript 3
ATF-6: Activating Transcription Factor 6
BIP: Immunoglobin Heavy Chain-binding Protein
HBSS: Hank’s Balanced Salt Solution
P/S: Penicillin & Streptomycin
CO_2_: Carbon Dioxide
CGM: Complete Growth Medium
FBS: Foetal Bovine Serum
PBS: Phosphate Buffered Saline
BSA: Bovine Serum Albumin
EqASCs_HE_: Healthy Adipose-Derived Stromal Stem Cells
EqASCs_EM__S_ 50 nM SHBG: EMS Affected Adipose-Derived Stromal Stem Cells Treated with 50 nM of SHBG for 24 h
EqASCs_EM__S_ 100 nM SHBG: EMS Affected Adipose-Derived Stromal Stem Cells Treated with 100 nM of SHBG for 24 h
7-AAD: 7-Aminoactinomycin D
RNAse: Ribonuclease H
PI: Propidium Iodide
NO: Nitric Oxide
DHE: Dihydroethidium
ERK: Extracellular Signal-Regulated Kinases
PFA: Paraformaldehyde
DAPI: 4',6-Diamidino-2-phenylindole
RIPA: Radioimmunoprecipitation
BCA: Bicinchoninic Acid
SDS-PAGE: Sodium Dodecyl-sulfate Polyacrylamide Gel Electrophoresis
PVDF: Polyvinylidene Difluoride
TBST: Tris-Buffered Saline Tween
HRP: Chemiluminescent and Fluorescent Peroxidase
mRNA: Messenger Ribonucleic Acid
miRNA: Micro-RNA
RT-qPCR: Reverse Transcription Quantitative Real-time PCR
gDNA: Genomic DNA
eiF2a: Eukaryotic Translation Initiation Factor 2A
cebpa: CCAAT Enhancer Binding Protein Alpha
PPARγ: Peroxisome Proliferator Activated Receptor Gamma
SOD1: Superoxide Dismutase 1
SOD2: Superoxide Dismutase 2
INSR: Insulin Receptor
IRS-1: Insulin Receptor Substrate 1
GAPDH: Glyceraldehyde-3-phosphate Dehydrogenase
CAT: Catalase
GPx: Glutathione Peroxidase
CHOP: DNA Damage Inducible Transcript 3
PERK: Eukaryotic Translation Initiation Factor 2 Alpha Kinase 3
IR: Insulin Resistance
Bad: BCL2 Associated Agonist Of Cell Death
Bcl-2: B-cell Lymphoma 2
Bcl-XL: B-cell lymphoma-extra large
Bax: Bcl-2-associated X protein
MDA: Malondialdehyde
ox-LDL: Oxidized Low-Density Lipoprotein
HDL: High-Density Lipoprotein
GSH: Glutathione
GRP78: Glucose-Regulated Protein 78
PIP3: Phosphatidylinositol (3,4,5)-trisphosphate
PKB: Protein Kinase B
PDK1: Phosphatidylinositol-Dependent Protein Kinase-1
GLUT1: Glucose Transporter 1
CREBA: Cyclic AMP Response Element-Binding Protein A
