# Supplementary material for: Sex hormone-binding globulin (SHBG) mitigates ER stress and improves viability and insulin sensitivity in adipose-derived mesenchymal stem cells (ASC) of equine metabolic syndrome (EMS)-affected horses
Source: Cell Commun Signal. 2023 Sep 11;21:230. doi: 10.1186/s12964-023-01254-6 (PMC10496240; doi:10.1186/s12964-023-01254-6)
Supplement: Supplementary file 3 — Additional file 2: Figure 1. Full-length blot for INSR protein. Figure 2. Full-length blot for IRS-1 protein. Figure 3. Full-length blot for Glut-4 protein. Figure 4. Full-length blot for β-Actin protein. [file 12964_2023_1254_MOESM2_ESM.docx]

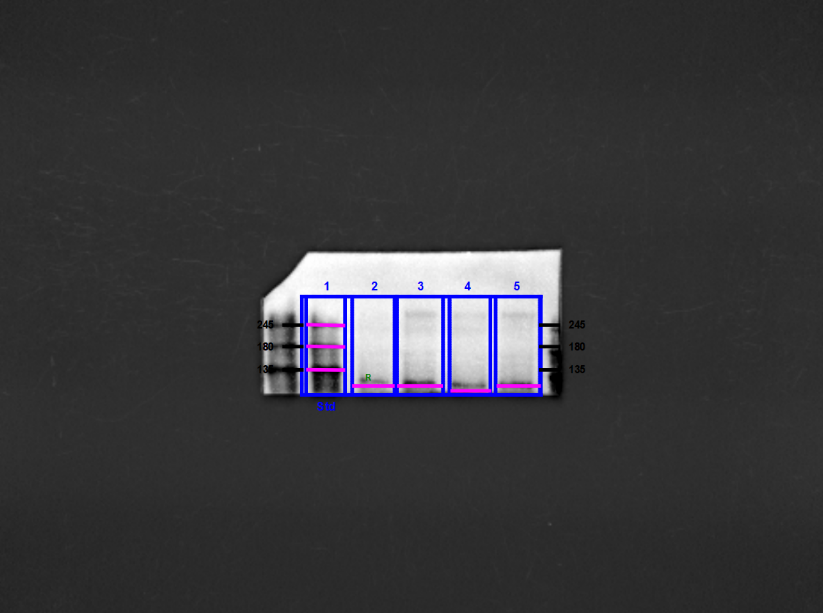


**Figure 1:** Full-length blot for INSR protein.


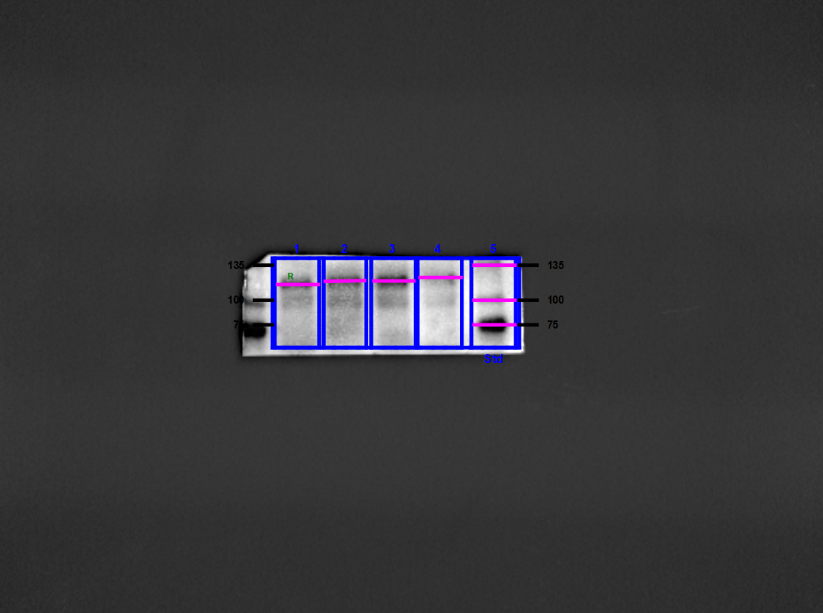


**Figure 2:** Full-length blot for IRS-1 protein.


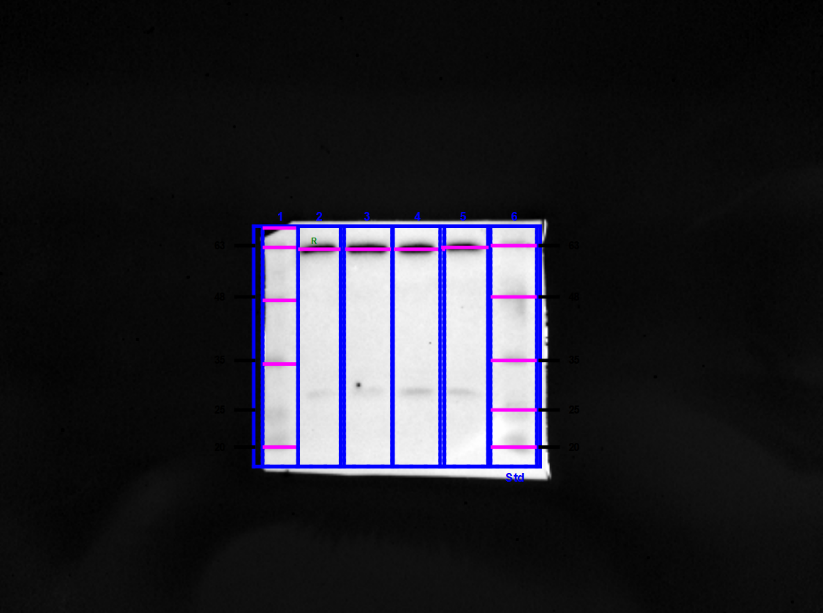


**Figure 3:** Full-length blot for Glut-4 protein.


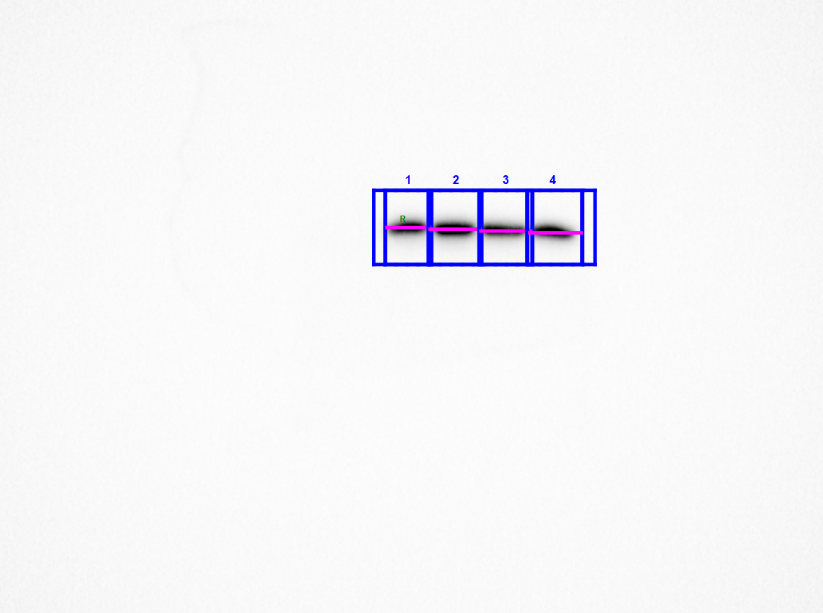


**Figure 4:** Full-length blot for β-Actin protein.
